# Male pheromone composition depends on larval but not adult diet in *Heliconius melpomene*

**DOI:** 10.1111/een.12716

**Published:** 2019-01-16

**Authors:** Kathy Darragh, Kelsey J. R. P. Byers, Richard M. Merrill, W. Owen McMillan, Stefan Schulz, Chris D. Jiggins

**Affiliations:** ^1^ Department of Zoology University of Cambridge Cambridge U.K.; ^2^ Smithsonian Tropical Research Institute Panama; ^3^ Division of Evolutionary Biology Ludwig‐Maximilians‐Universität Munich Germany; ^4^ Department of Life Sciences, Institute of Organic Chemistry, Institute of Organic Chemistry Technische Universität Braunschweig Braunschweig Germany

**Keywords:** Chemical signalling, effect of diet, host plant, Lepidoptera, mate choice, pollen feeding, sexual selection

## Abstract

1. Condition‐dependent traits can act as honest signals of mate quality, with fitter individuals being able to display preferred phenotypes. Nutrition is known to be an important determinant of individual condition, with diet known to affect many secondary sexual traits.

2. In *Heliconius* butterflies, male chemical signalling plays an important role in female mate choice. Potential male sex pheromone components have been identified previously, although it is unclear what information they convey to the female.

3. In the present study, the effect of diet on androconial and genital compound production is tested in male *Heliconius melpomene rosina*. To manipulate larval diet, larvae are reared on three different *Passiflora* host plants: *Passiflora menispermifolia*, the preferred host plant, Passiflora vitifolia and Passiflora platyloba. To manipulate adult diet, adult butterflies are reared with and without access to pollen, a key component of their diet.

4. No evidence is found to suggest that adult pollen consumption affects compound production in the first 10 days after eclosion. There is also a strong overlap in the chemical profiles of individuals reared on different larval host plants. The most abundant compounds produced by the butterflies do not differ between host plant groups. However, some compounds found in small amounts differ both qualitatively and quantitatively. Some of these compounds are predicted to be of plant origin and the others synthesised by the butterfly. Further electrophysiological and behavioural experiments will be needed to determine the biological significance of these differences.

## Introduction

Sexual ornaments often act as an indicator of mate quality and evolve in response to sexual selection imposed by female preferences (Zahavi, 1975, 1977; Andersson, 1986). Male ‘quality’ can reflect both direct and indirect benefits gained by the female (Andersson, 1994). Direct benefits might include resources that increase female lifetime reproductive success, such as food, shelter, parental care or protection from predators. Indirect benefits, on the other hand, are those that increase genetic quality of a female's offspring. In this case, sexually selected traits reflect the ability of males to provide genes that increase the survivorship or mating success of offspring (Andersson, 1986, 1994). These traits may be an honest signal of quality if they are condition‐dependent, where only the best quality males are able to display the phenotype.

Male pheromones are a good candidate as an honest signal. Diet‐mediated changes can enforce signal reliability (Henneken *et al*., 2017) and compounds can be costly to produce (Johansson *et al*., 2005; Harari *et al*., 2011). Nutritional condition affects male pheromone production in *Tenebrio* beetles (Rantala *et al*., 2003), cockroaches (Clark *et al*., 1997) and burying beetles (Chemnitz *et al*., 2015). Diet manipulation studies show that diet not only affects pheromone production, but also that these changes affect female mate choice (e.g. cockroaches: South et al., 2011; fruit flies: Liedo et al., 2013).

In addition to overall diet quality, specific diet components can be important. Compounds sequestered in the diet can act directly as sex pheromones or as pheromone precursors that are then metabolised further (Landolt & Phillips, 1997). One well‐studied example of this is the sequestration of pyrrolizidine alkaloids (PAs) to make hydroxydanaidal by males of the moth *Utethesia ornatrix* (Conner *et al*., 1981; Eisner & Meinwald, 2003). Some PAs are transferred to the female during mating and chemically protect the eggs, with the male pheromone signalling a direct benefit (Dussourd *et al*., 1988, 1991; Eisner & Meinwald, 1995; Iyengar *et al*., 2001). In many cases, it is probable that both overall nutrient condition and the consumption of specific compounds are important, such as in the oriental fruit fly, where both overall protein intake and the intake of a specific precursor, methyl eugenol, affect mating success (Shelly *et al*., 2007).

Diet shifts can provide species with new ecological and evolutionary opportunities. Being unique among butterflies, *Heliconius* are able to feed on pollen (Gilbert, 1972). They collect pollen from flowers and masticate it on their proboscis to extract amino acids. *Heliconius* have a long lifespan in the wild, facilitated by pollen feeding, which is important for oviposition and viability (Dunlap‐Pianka *et al*., 1977). The lack of dependence upon larval resources for reproduction may have facilitated a greater investment in defensive compounds during the larval stage (Cardoso & Gilbert, 2013). As larvae, *Heliconius* caterpillars feed on the cyanogenic leaves of *Passiflora*. *Heliconius* butterflies produce cyanogenic compounds *de novo*, making them unpalatable, and some are also able to sequester compounds directly from *Passiflora* plants (Engler‐Chaouat & Gilbert, 2006). Both larval and adult diet play important roles in *Heliconius* biology, affecting reproductive lifespan and palatability.

The importance of diet for chemical signalling in *Heliconius* is unclear. The role of chemical signalling in mate choice has been best studied in *Heliconius melpomene rosina* Boisduval (Nymphalidae), a subspecies of *Heliconius melpomene* found in central Panama. Potential male sex pheromone components have been described in the wing overlap region of sexually mature males (Darragh *et al*., 2017). The morphology of this wing region is sexually dimorphic, with specialised androconial scales only found on male wings. A bouquet of compounds that include octadecanal as a main component is found in males but not females (Mérot *et al*., 2015; Darragh *et al*., 2017; Mann *et al*., 2017). These chemical cues are important for mating, with females strongly discriminating against males that have their androconia experimentally blocked (Darragh *et al*., 2017). However, it is still unclear what information (e.g. age or male quality) is being conveyed to females by these cues.

By contrast to androconial compounds, which are assumed to be aphrodisiac in nature, *Heliconius* males also store anti‐aphrodisiac compounds in genital scent glands (Gilbert, 1976; Schulz *et al*., 2007, 2008; Estrada *et al*., 2011). These are transferred to the female and repel males, delaying re‐mating (Gilbert, 1976; Schulz *et al*., 2008). In *H. melpomene*, (*E*)‐β‐ocimene acts as an anti‐aphrodisiac (Schulz *et al*., 2008). Reduced harassment by other males is considered to be beneficial to the female, and so these compounds could lead to a direct benefit to females. Longer‐term, there may be conflict over the timing of re‐mating, as supported by the rapid evolution of genital chemical composition (Andersson *et al*., 2000, 2004; Estrada *et al*., 2011). Despite this clear role of genital compounds in male deterrence, the role of these same compounds in female choice remains unknown. Females may benefit from choosing males that have a lower amount of (*E)*‐β‐ocimene, allowing them to re‐mate again sooner. Although the dynamics of the costs and benefits of this are unclear, it is quite likely that the genital compounds are involved in female choice.

Both larval and adult diet could be important for the production of androconial and genital compounds because they are not present in freshly‐eclosed males (Schulz *et al*., 2008; Darragh *et al*., 2017). Feeding experiments with chemically labelled precursors showed that the anti‐aphrodisiac compound, (*E*)‐β‐ocimene, can be synthesised by adult *H. melpomene* via the terpene biosynthetic pathway (Schulz *et al*., 2008). Pollen intake could be important in providing an energy source for production. Host plant use could affect the chemical bouquet if larval sequestration of specific compounds, or compound precursors, from the host plant is necessary. *Heliconius* raised on their preferred host plant may have a higher quality diet (Smiley, 1978) and so compound production could also be increased as a result of higher overall quality of the individual.

In the present study, we investigated how larval and adult diet affect the chemical profile of male *H. melpomene rosina* from central Panama. In Panama, *H. melpomene rosina* females oviposit almost exclusively on *Passiflora menispermifolia* (Merrill *et al*., 2013). We reared larvae on three different *Passiflora* species: *Passiflora menispermifolia*, the preferred host plant, *Passiflora vitifolia* and *Passiflora platyloba*. The latter two species are not used by *H. melpomene* in the wild in Panama but are found within the range of *H. melpomene rosina*. These species are therefore potential hosts and larvae survive well on both (Merrill *et al*., 2013). *Heliconius melpomene* reared on its preferred host plant may have increased energy sources to dedicate to compound production during adult life. We predict the existence of both qualitative and quantitative differences in the chemical bouquets of adults reared on different host plants. In a second experiment, we maintained adult male *H. melpomene rosina* with and without access to pollen. We predict that males reared without pollen demonstrate reduced compound production. In both experiments, we analysed chemical extracts from both the androconial and genital regions of sexually mature male butterflies.

## Materials and methods

### *Butterfly stocks*

*Heliconius melpomene rosina* were reared under ambient conditions at the Smithsonian Tropical Research Institute facilities in Gamboa, Panama. Outbred stocks were established from wild individuals collected in Gamboa (9°7.4′N, 79°42.2′W, elevation 60 m) in the nearby Soberania National Park and in San Lorenzo National Park (9°17′N, 79°58′W; elevation 130 m). Individuals for the study were reared between February 2016 and April 2017.

### *Effects of larval diet*

Larvae were reared on either *P. platyloba*, *P. vitifolia* or *P. menispermifolia* (preferred host plant). Adult butterflies were kept in cages with other males and were provided with an approximately 20% sucrose solution containing bee pollen (Apiarios Malivern, Panama) and with *Psychotria poeppigiana*, *Gurania eriantha*, *Psiguiria triphylla* and *Psiguria warscewiczii* as pollen sources. We collected 42 androconial samples from adult butterflies: 19 reared on *P. platyloba*, 11 on *P. menispermifolia* and 12 on *P. vitifolia*. We also collected 43 genital samples from adult butterflies: 17 reared on *P. platyloba*, 13 on *P. menispermifolia* and 13 on *P. vitifolia*. We aimed for a minimum of 10 individuals in each group. Variance between groups was a result of the availability of host plants during the experiment and rearing difficulties on different host plants. Variance between the number of androconial and genital samples within each group is a result of issues with contamination of samples, which were then unsuitable for analysis. The 19 androconial samples from larvae reared on *P. platyloba* have been described previously (Darragh *et al*., 2017).

To account for a potential difference in growth rate of individuals reared on different host plants, we measured the forewing length of adult butterflies for the host plant experiments. Before cutting the wings for chemical analysis, we photographed wings beside a ruler. We used imagej (NIH, Bethseda, Maryland) to calculate forewing length, calibrating the size using the ruler (Schneider *et al*., 2012).

### *Effects of adult diet*

All larvae were reared on *P. platyloba* because this was the plant that was available in the largest quantities. Adults were then randomly divided into two groups. The first was provided with an approximately 20% sugar solution containing bee pollen (Apiarios Malivern, Panama) and *P. poeppigiana*, *G. eriantha*, *P. triphylla* and *P. warscewiczii* as pollen sources; the second group was only provided with an approximately 20% sugar solution and no pollen source. We analysed the androconia of 20 individuals reared with pollen and 33 without, as well as the genitals of 20 individuals reared with pollen and 27 without.

### *Extraction and chemical analysis of tissues*

Chemical extractions were carried out on androconial and genital tissue of mature male individuals (10–12 days post‐eclosion). The individuals raised on *P. platyloba* for the host plant experiment are previously reported samples (Darragh *et al*., 2017). Genitals were removed using forceps. The wings of the individual were then removed. The hindwing androconial region of the wing, previously described as the grey–brown overlapping region of the wing (Darragh *et al*., 2017), was dissected from the wings for analysis. To extract compounds, the tissue was soaked, immediately after dissection, in 200 µl of dichloromethane containing 200 ng of 2‐tetradecyl acetate (internal standard) in 2‐ml glass vials with polytetrafluoroethylene‐coated caps (Agilent, Santa Clara, California) for 1 h. The solvent was then transferred to new vials and stored at −20 °C. Samples were evaporated in the laboratory at room temperature prior to analysis.

Chemical extracts were analysed by gas chomatography‐mass spectrometry (GC‐MS) using an Agilent (model 5977) mass‐selective detector connected to an Agilent GC (model 7890B). This was equipped with an Agilent ALS 7693 autosampler and an HP‐5MS fused silica capillary column (Agilent) (length 30 m, inner diameter 0.25 mm, film thickness 0.25 μm). Injection was performed in splitless mode (injector temperature 250 °C) with helium as the carrier gas (constant flow of 1.2 ml min^−1^). The temperature programme started at 50 °C, was held for 5 min, and then rose at a rate of 5 °C min^−1^ to 320 °C, before being held at 320 °C for 5 min. Components were identified by comparison of mass spectra and gas chromatographic retention indices with those of authentic reference samples and also by analysis of mass spectra. Components were quantified using 2‐tetradecyl acetate as an internal standard. Only compounds eluting earlier than hexacosane were analysed in androconial samples (Darragh *et al*., 2017). Later compounds were identified as cuticular hydrocarbons, 2,5‐dialkyltetrahydrofurans, cholesterol and artefacts (e.g. phthalates or adipates). The variability in the late eluting cuticular hydrocarbons was low and did not show characteristic differences between samples.

### *Statistical analysis*

We visualised the data using a non‐metric multidimensional scaling (NMDS) ordination, based on a Bray–Curtis similarity matrix, in three dimensions. This was carried out using the metaMDS function in the r‐package vegan, version 2.5‐1 (Oksanen *et al*., 2017), with visualisation using the r‐package ade4 (Dray & Dufour, 2007).

After visualisation of the data, we used multivariate statistical techniques to investigate differences between the groups. To identify differences in variance between groups, we used the betadisper and permutest functions to test for homogeneity of dispersion. To compare overall chemical composition between groups, we carried out permutational multivariate analysis of variance (permanova) testing. This was performed using a Bray–Curtis similarity matrix, with 1000 permutations, using the adonis2 function in the package vegan (Oksanen *et al*., 2017). The ‘margin’ option in adonis2 was used to determine the effect of each term in the model, including all other variables, aiming to avoid sequential effects. The dependent variable in the model was the matrix of compounds, with different explanatory variables in the two experiments. In the larval diet experiments, we included wing length and plant species as explanatory variables. In the adult diet experiment, we tested the explanatory variable of presence or absence of pollen. We repeated the multivariate analysis using relative rather than absolute amounts of compounds.

We followed up the multivariate statistical analysis with univariate analysis to test for differences in amounts of individual compounds between groups. We tested each compound individually using non‐parametric Kruskal–Wallis tests. To correct for multiple‐testing, we used the p.adjust function in r, with false detection rate correction, which controls for the proportion of false positives. We repeated the univariate analysis using relative rather than absolute amounts of compounds.

To test for differences in forewing length for individuals reared on different host plants we carried out an anova. For *post hoc* analysis, Tukey's honestly significant difference test was used to determine which groups were significantly different from each other. All statistical analyses were performed with r, version 3.3.1 (R Core Team, 2016).

## Results

### *Effect of host plant on wing size*

**Figure 1:**
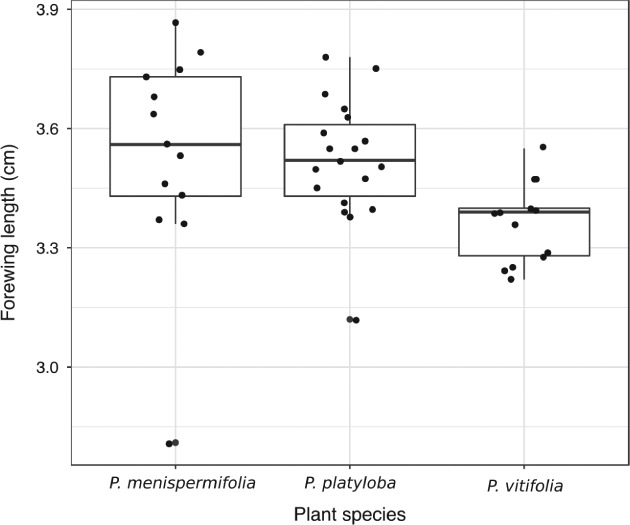
Larval host plant affects forewing length of *Heliconius melpomene* male adults (anova, d.f. = 2, *F* = 3.755, *P* = 0.032). *Post hoc* testing using Tukey's honestly significant difference found no significant pairwise differences between groups.

Larval host plant affected forewing length (anova, *F*_2,42_ = 3.755, *P* = 0.032) (Fig. 1). Mean ± SD forewing length was 3.54 ± 0.27 cm for adults reared as larvae on *P. menispermifolia* (the preferred host plant of *H. melpomene rosina*), 3.52 ± 0.15 cm for those reared on *P. platyloba* and 3.36 ± 0.10 cm for those reared on *P. vitifolia*. However, *post hoc* Tukey comparisons did not find any pairwise significant difference between groups.

### *Chemical compounds in androconia and genitals of* H. melpomene

We initially analysed 19 androconia samples and 18 genital samples of *H. melpomene* reared on *P. platyloba*. This is a reanalysis of the 19 androconial samples that have been described previously (Darragh *et al*., 2017). The most abundant compounds found in the androconia are syringaldehyde, octadecanal, octadecan‐1‐ol, (*Z*)‐11‐icosenal and (Z)‐11‐icosenol, as reported previously (see Supporting information, Figure S1 and Table S1) (Darragh *et al*., 2017). These compounds are present with a mean of greater than 100 ng per individual, with octadecanal being found in the highest amounts (mean 740 ng).

The genital region is dominated by one main compound, (*E*)‐β‐ocimene, which is found in 20‐fold greater amounts than any other genital compound (mean 34789 ng). This was previously reported in other samples of *H. melpomene* (Schulz *et al*., 2008). It is found alongside a bouquet of other terpenes, alcohols, aromatic compounds, macrolides, esters and alkanes (see Supporting information, Figure S2 and Table S2) (Schulz *et al*., 2008).

There is little overlap in compounds found between the two body regions, with only 10 out of 117 compounds being found in both. The genital region contains higher amounts of compounds and more compounds overall, with 80 in the genitals compared with 47 in the androconia. The most abundant genital compound, (*E*)‐β‐ocimene, is more volatile (has a higher vapour pressure) than the main compounds found in the androconial region.

### *Effects of larval diet*

Wing size was not a significant factor influencing chemical composition of *H. melpomene* genitals (permanova, *F*_1,39_ = 0.577, *P* = 0.606) but did influence androconial chemical bouquets (permanova, *F*_1,38_ = 3.033, *P* = 0.038), accounting for approximately 7% of the variation.

**Figure 2:**
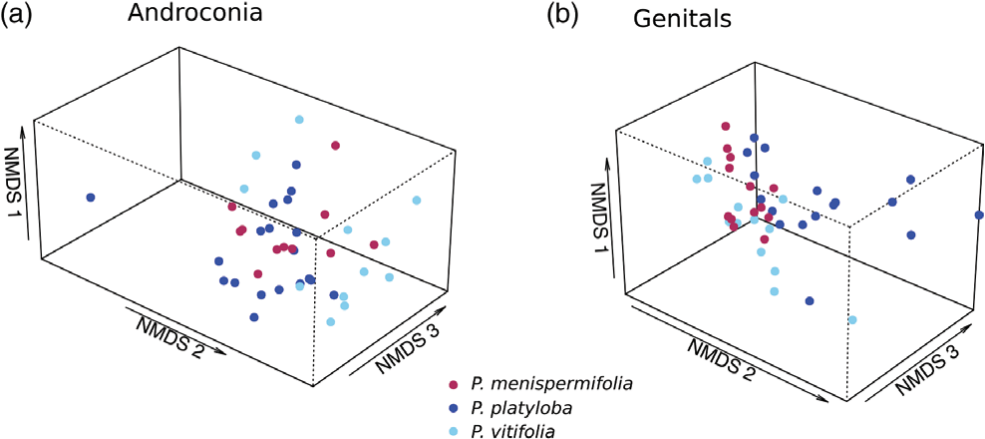
Non‐metric multidimensional scaling (NMDS) plot illustrating in three dimensions the overlapping variation in chemical compounds of male *Heliconius melpomene* raised on three different *Passiflora* species. *Passiflora menispermifolia* is the preferred host plant of this species. (a) Androconial compound bouquets do not differ significantly after 10 days. Stress = 0.140. (b) Genital compound bouquets do not differ significantly after 10 days. Stress = 0.098. [Colour figure can be viewed at wileyonlinelibrary.com].

Our experiments revealed that *H. melpomene* reared on *P. platyloba*, *P. menispermifolia* or *P. vitifolia* did not differ significantly in their overall androconial bouquet (permanova, *F*_2,38_ = 1.791, *P* = 0.080) (Fig. 2a; see also Supporting information, Table S1) and did not differ in dispersion between groups (permutation test of homogeneity of dispersion, *F*_2,39_ = 1.335, *P* = 0.275). However, when we look at the individual compounds in each treatment, more than one‐quarter (12/47) are present in significantly different amounts between groups (Table 1). These same compounds were also found to be significantly different between groups using relative amounts (for further analysis of relative amounts, see Supporting information).

**Table 1:** Androconial compounds that significantly differed between *Heliconius melpomene* reared on different host plants.

Chemical	RI	*Passiflora platyloba*	*Passiflora menispermifolia*	*Passiflora vitifolia*	*H* test statistic	*P*‐value
Methyl salicylate	1189	0.59 ± 1.97	0.98 ± 3.13	2.32 ± 1.95	10.23	0.026
Unknown compound	1353	0 ± 0	0.40 ± 1.31	3.30 ± 3.85	16.68	0.006
**1‐(3,5‐Dimethoxy‐4‐hydroxybenzyl)ethanone**	1735	0.99 ± 1.58	0.67 ± 0.76	0.01 ± 0.04	10.71	0.022
Benzyl benzoate	1766	0.25 ± 0.57	0.13 ± 0.44	1.27 ± 1.40	10.78	0.022
**1‐(4‐Hydroxy‐3,5‐dimethoxyphenyl)‐2‐propen‐1‐one**	1807	1.48 ± 1.93	1.15 ± 1.32	0 ± 0	12.06	0.016
**Syringaldehyde derivative**	1891	0 ± 0	3.05 ± 2.74	0 ± 0	19.11	0.003
Unknown hydrocarbon	1962	1.32 ± 1.14	1.80 ± 1.41	0.28 ± 0.97	11.19	0.022
**Ethyl 4‐hydroxy‐3,5‐ dimethoxybenzoate**	2057	32.61 ± 55.79	11.60 ± 13.95	0.14 ± 0.37	13.52	0.011
Henicosadiene	2065	1.36 ± 4.28	0.22 ± 0.74	2.97 ± 3.91	9.75	0.030
Methyloctadecanal	2077	30.77 ± 15.99	15.37 ± 8.54	10.94 ± 13.15	13.92	0.011
Icosanal	2224	23.21 ± 11.46	16.08 ± 14.41	8.41 ± 7.59	14.92	0.009
Fatty acid amide	2325	0.27 ± 1.20	0 ± 0	1.09 ± 1.66	12.53	0.015

The gas chromatographic retention index (RI) is reported for each compound. Mean ± SD amounts (ng), as well as Kruskal–Wallis non‐parametric test *P*‐values (false detection rate corrected) are provided. Compounds shown in bold are predicted to be plant‐derived.

Genital compounds of *H. melpomene* reared on *P. platyloba*, *P. menispermifolia* or *P. vitifolia* did not differ overall between host plant treatments (permanova, *F*2,39 = 1.184, *P* = 0.308) (Fig. 2b; see also Supporting information, Table S2). The dispersion of individuals between treatments did differ (permutation test of homogeneity of dispersion, *F*_2,40_ = 3.668, *P* = 0.034), with pairwise permutation analysis revealing that the dispersion of individuals raised on *P. menispermifolia* is different from both *P. vitifolia* and *P. platyloba*, which do not differ from each other (see Supporting information, Table S3). NMDS visualisation reveals less variation between individuals reared on the preferred host *P. menispermifolia* (Fig. 2b). Furthermore, seven out of 80 individual compounds differ between groups (Table 2). These compounds were also found to be significantly different between groups using relative amounts, excluding an unknown compound (gas chromatographic retention index, RI = 1396), which is no longer significant (for further analysis of relative amounts, see Supporting information).

**Table 2:** Genital compounds that differed significantly between *Heliconius melpomene* reared on different host plants.

Chemical	RI	*Passiflora platyloba*	*Passiflora menispermifolia*	*Passiflora vitifolia*	*H* test statistic	*P*‐value
**7‐β‐(H)‐Silphiperfol‐5‐ene**	1345	9.75 ± 12.23	0 ± 0	0 ± 0	21.46	< 0.001
**Unknown sesquiterpene**	1378	2.87 ± 4.20	0 ± 0	0 ± 0	19.02	0.001
**Unknown sesquiterpene**	1384	20.90 ± 21.99	0 ± 0	0 ± 0	32.40	< 0.001
Unknown compound	1396	39.76 ± 26.86	26.83 ± 30.96	8.23 ± 6.87	16.03	0.004
**β‐Caryophyllene**	1417	18.97 ± 22.09	0 ± 0	0 ± 0	32.40	< 0.001
Unknown compound	2250	0 ± 0	1.48 ± 5.32	1.33 ± 2.09	11.37	0.039
Cholestadiene	2744	0 ± 0	9.56 ± 34.48	18.18 ± 24.10	19.96	< 0.001

The gas chromatographic retention index (RI) is reported for each compound. Mean ± SD amounts (ng), as well as Kruskal–Wallis non‐parametric test *P*‐values (false detection rate corrected) are provided. Compounds shown in bold are predicted to be plant‐derived.

None of the individual compounds found to differ between groups were the most abundant compounds. These compounds are not found in an average amount higher than 40 ng per individual, which is much smaller amount than the most abundant compound in androconia (740 ng) or genitals (34 789 ng). We found both qualitative (presence or absence of compound) and quantitative (difference in amount of compound) differences between butterflies reared on different host plants. For example, in the androconial samples, a syringaldehyde derivative was only found in individuals reared on *P. menispermifolia*. Icosanal, in contrast, was found in butterflies reared on all three host plant species but in significantly different amounts (Table 1).

### *Effects of adult diet*

**Figure 3:**
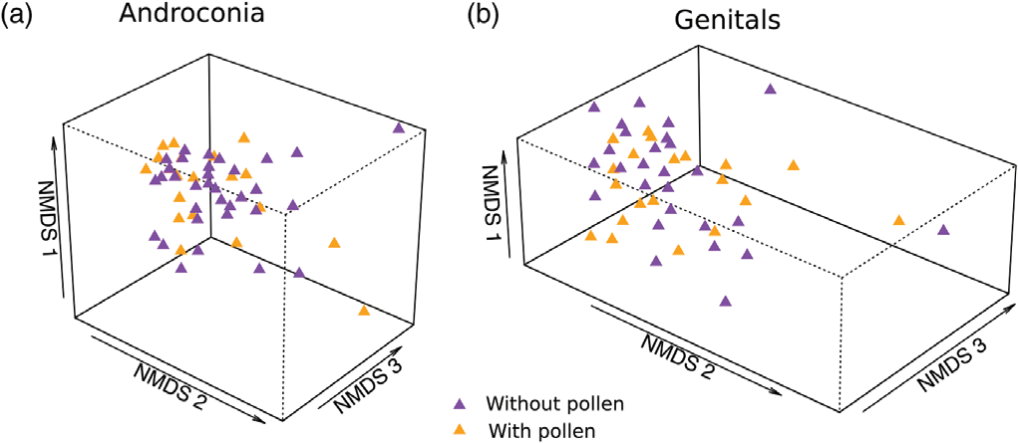
Non‐metric multidimensional scaling (NMDS) plot illustrating in three dimensions the overlapping variation in chemical compounds of male *Heliconius melpomene* raised with or without pollen. (a) Androconial compound bouquets do not differ significantly after 10 days. Stress = 0.131. (b) Genital compound bouquets do not differ significantly after 10 days. Stress = 0.108. [Colour figure can be viewed at wileyonlinelibrary.com].

*Heliconius melpomene* butterflies reared with or without pollen for 10 days do not differ in either androconial (permanova, *F*_1,51_ = 1.653, *P* = 0.145) or genital (permanova, *F*_1,45_ = 1.259, *P* = 0.260) chemical bouquets (Fig. 3; see also Supporting information, Tables S4 and S5). False detection rate corrected Kruskal–Wallis testing found no compounds in significantly different amounts between the groups.

## Discussion

Chemical signalling is known to be important for intraspecific female mate choice in *Heliconius* (Darragh *et al*., 2017). The information conveyed by these compounds, such as age, species identity or mate quality, remains unclear. We find no evidence that adult pollen consumption affects compound production in the first 10 days of adult life. By contrast, individual androconial and genital compounds were found in different amounts between larval host plant treatment groups, and dispersion varied between host plant treatments for genitals.

The most abundant compounds identified in the androconia and genitals of *H. melpomene* are the same as those identified in previously published studies (Schulz *et al*., 2008; Estrada *et al*., 2011; Mérot *et al*., 2015; Darragh *et al*., 2017; Mann *et al*., 2017). We did not identify all of the compounds found previously in genitals (Schulz *et al*., 2008). This is likely a result of variation in sample collection because the list of previous compounds was derived from pooled samples, allowing for a higher detection threshold. In the androconia, we did not detect ethyl palmitate, ethyl oleate or ethyl stearate, previously reported compounds (Mann *et al*., 2017). We did detect ethyl oleate in the genitals, suggesting that previous reports in the androconia may be a result of contamination from genital contact. We also found many more compounds, probably because of improved GC‐MS detection thresholds that are more sensitive to compounds found only in low levels.

We did not find a difference in the ability of males to produce compounds when reared with and without pollen in this experiment. This finding was somewhat unexpected because pollen is an important resource for adult *Heliconius*. This result could mean that chemical signalling is not nutritionally dependent. *Heliconius* comprise one of the most long‐lived butterflies, with adults being known to live more than 8 months in the wild (Gilbert, 1972), and pollen limitation might play a more important role over longer time scales. In females, the effects of pollen for oviposition and viability are evident after approximately 1 month (Dunlap‐Pianka *et al*., 1977), suggesting that, until that point, larval reserves are sufficient. This could be the same for males and therefore we cannot rule out the possibility that adult nutrition might influence pheromone production only later in life.

Pollen‐feeding is considered to be important for another aspect of reproduction in *Heliconius*: the donation of a spermatophore to the female during mating. The spermatophore is protein‐rich, and so the amino acids obtained by pollen‐feeding are probably needed to make new spermatophores after each mating (Cardoso & Silva, 2015), as supported by the finding that males with more lifetime matings collect more pollen overall (Boggs, 1990). This is beneficial for the females because its reduces the need for females to forage for pollen (Boggs & Gilbert, 1979; Boggs, 1981, 1990). It has been proposed that females may determine spermatophore quality using cues (e.g. chemicals) that indicate direct benefits for the female (Cardoso & Silva, 2015). Alternatively, females could benefit indirectly through a ‘good genes’ mechanism (Andersson & Simmons, 2006); for example, through inheritance of foraging ability (Karino *et al*., 2005). Further experiments will be required to determine whether chemical signalling in older males can indicate spermatophore production and male quality.

Chemical profiles produced by adult butterflies reared on the three larval host plants are largely overlapping. *Heliconius melpomene* is able to produce the majority of compounds found in both androconial and genital bouquets when reared on all three *Passiflora* species. The most abundant compounds are not found in significantly different amounts. However, we find less variation in genital compounds produced by individuals reared on *P. menispermifolia*, the preferred host plant of *H. melpomene rosina*, compared with *P. vitifolia* and *P. platyloba*, perhaps suggesting some level of chemical or digestive specialisation.

Despite an overall similarity between butterflies reared on different host plants, there are differences in some specific androconial and genital compounds. These differences are both qualitative and quantitative in nature. Over one‐quarter of androconial compounds are found in significantly different amounts between the three groups, along with almost one‐tenth of genital compounds. One‐third of these significant androconial compounds are considered to come from the phenylpropanoid pathway, active in plants (Boerjan *et al*., 2003). This pathway forms aromatic compounds with an alkyl sidechain of three carbons that serve as building blocks for lignin and lignans. Oxidative degradation of lignin or by‐products of this biosynthetic pathway are the source of the aromatic compounds syringaldehyde, 1‐(3,5‐dimethoxy‐4‐hydroxybenzyl)ethanone or ethyl 4‐hydroxy‐3,5‐dimethoxybenzoate. They are not currently known to act as pheromones in insects, although closely‐related compounds, lacking one methoxy group, are reported as fruit fly and moth pheromones (Francke & Schulz, 2010).

Over one‐half of the genital compounds, specifically sesquiterpenes, are also considered to originate from plant sources. These include the specific compounds 7‐β‐(H)‐silphiperfol‐5‐ene and β‐caryophyllene, as well as some unknown other sesquiterpenes. The genome currently does not show any indication of a required sesquiterpene cyclase in *H. melpomene*, thus making *de novo* synthesis of sesquiterpenes by the butterflies unlikely. These data suggest that differences in plant biochemistry affect the chemicals released from both androconial and genital regions of the adult butterfly.

We do not know which components of the androconial bouquet are biologically important in *H. melpomene*. It cannot be assumed that the most abundant compounds are necessarily the most important because minor compounds can often play important roles in attraction (D'Alessandro *et al*., 2009; McCormick *et al*., 2014). Furthermore, the response to pheromonal cues is blend‐specific in other Lepidopteran systems (Yildizhan *et al*., 2009; Larsdotter‐Mellström *et al*., 2016). Despite the overlap in overall chemical composition between host plant treatments, those compounds that are significantly different could possibly drive a change in female response. This is particularly likely for the androconial bouquet, where more than one‐quarter of compounds are found in different amounts between larval host plant treatments. However, it is important to note that direct tissue extraction of chemicals may not accurately reflect the chemical amounts emitted by live butterflies (Visser *et al*., 2018). Electrophysiological and behavioural experiments will be required to determine whether these differences are biologically relevant.

Our results contribute to an understanding of why some populations of *H. melpomene* are host specialists when their larvae can successfully feed on a wider variety of host plants. *Heliconius melpomene* larval growth rate is similar on different host plants under laboratory conditions (Smiley, 1978), although there are slight differences in survival in the wild, perhaps as a result of ant attendance or parasitism (Merrill *et al*., 2013). In particular, *H. melpomene* fed on *P. vitifolia* show a somewhat lower survival rate compared with the natural host plant *P. menispermifolia* (Merrill *et al*., 2013), which may be related to our finding that host plant affects size in *Heliconius melpomene*. If host specialisation is not a result of physiological adaptation in the larvae, an alternative is that it could be explained by sexual selection. Female choice on diet‐derived pheromones could potentially drive host plant specialisation (Quental *et al*., 2007). In this case, we would not expect larval host plant specialisation because selection is acting on the adult stage. To determine whether this could be a plausible mechanism to explain host plant preference in *Heliconius*, we would need to determine whether the compounds that change with host plant use are also important for female choice.

We might expect to find intraspecific differences in chemistry between *Heliconius* races. Across their geographical range, *Heliconius* butterflies use different host plants and can vary in their extent of host plant specialisation (Benson *et al*., 1975; Benson, 1978). Based on our results, we predict that this will lead to differences in chemical bouquets between populations as a result of differing host plant use. We might also expect to find more intraspecific differences in chemistry in populations that are more generalist. Future investigations using different geographical races will help us understand the role of diet in the chemistry of *Heliconius* butterflies, as well as its link to host plant specialisation.

## Supporting information


**Figure S1.** Total ion chromatogram of extract from androconial region of *Heliconius melpomene*.
**Figure S2.** Total ion chromatogram of extract from genital region of *Heliconius melpomene*.
**Table S1.** Androconial compounds identified in *Heliconius melpomene rosina* males reared on Passiflora platyloba, *Passiflora menispermifolia* or Passiflora vitifolia.
**Table S2.** Genital compounds identified in *Heliconius melpomene rosina* males reared on Passiflora platyloba, *Passiflora menispermifolia* or Passiflora vitifolia.
**Table S3.** Pairwise comparisons of dispersion of *Heliconius melpomene* genital compounds when reared on different plants.
**Table S4.** Androconial compounds identified in *Heliconius melpomene rosina* males fed as adults with or without pollen.
**Table S5.** Genital compounds identified in *Heliconius melpomene rosina* males fed as adults with or without pollen.
