# Supplementary material for: Male pheromone composition depends on larval but not adult diet in Heliconius melpomene
Source: Ecol Entomol. 2019 Jan 16;44(3):397–405. doi: 10.1111/een.12716 (PMC6563479; doi:10.1111/een.12716)
Supplement: Supplementary file 1 — Figure S1. Total ion chromatogram of extract from androconial region of Heliconius melpomene. Figure S2. Total ion chromatogram of extract from genital region of Heliconius melpomene. Table S1. Androconial compounds identified in Heliconius melpomene rosina males reared on Passiflora platyloba, Passiflora menispermifolia or Passiflora vitifolia. Table S2. Genital compounds identified in Heliconius melpomene rosina males reared on Passiflora platyloba, Passiflora menispermifolia or Passiflora vitifolia. Table S3. Pairwise comparisons of dispersion of Heliconius melpomene genital compounds when reared on different plants. Table S4. Androconial compounds identified in Heliconius melpomene rosina males fed as adults with or without pollen. Table S5. Genital compounds identified in Heliconius melpomene rosina males fed as adults with or without pollen. [file EEN-44-397-s001.docx]

Supporting Information

**Results. Repeat of analyses on relative compound amounts in samples. Results found were similar to analysis of absolute compound amounts.**

We repeated the analysis on the relative rather than absolute compound amounts. We found that *H. melpomene* reared on *P. platyloba, P. menispermifolia*, or *P. vitifolia* did differ significantly in their overall androconial bouquet (PERMANOVA, F_2,38_=2.015, p=0.048). This was not detected using absolute amounts, however, it only accounts for 9% of variation. We did not detect a difference in dispersion between groups (permutation test of homogeneity of dispersion, F_2,39_=0.559, p=0.559). All the same compounds found in significantly different amounts were the same as those found using absolute amounts (Table 1).

We found that *H. melpomene* reared on *P. platyloba, P. menispermifolia*, or *P. vitifolia* did not differ significantly in their overall genital compound bouquet (PERMANOVA, F_2,39_=0.592, p=0.797). In contrast to the absolute amount analysis, we did not detect a difference in dispersion between groups (permutation test of homogeneity of dispersion, F_2,40_=1.254, p=0.296). The same compounds found in significantly different amounts were the same as those found using absolute amounts (Table 2), apart from the unknown compound (RI=1396) which is no longer significant.

As found with analysis of absolute amounts, *H. melpomene* butterflies reared with or without pollen for 10 days do not differ in either androconial (PERMANOVA, F_1,51_=2.235, p=0.063), or genital (PERMANOVA, F_1,45_=0.795, p=0.500) bouquets.


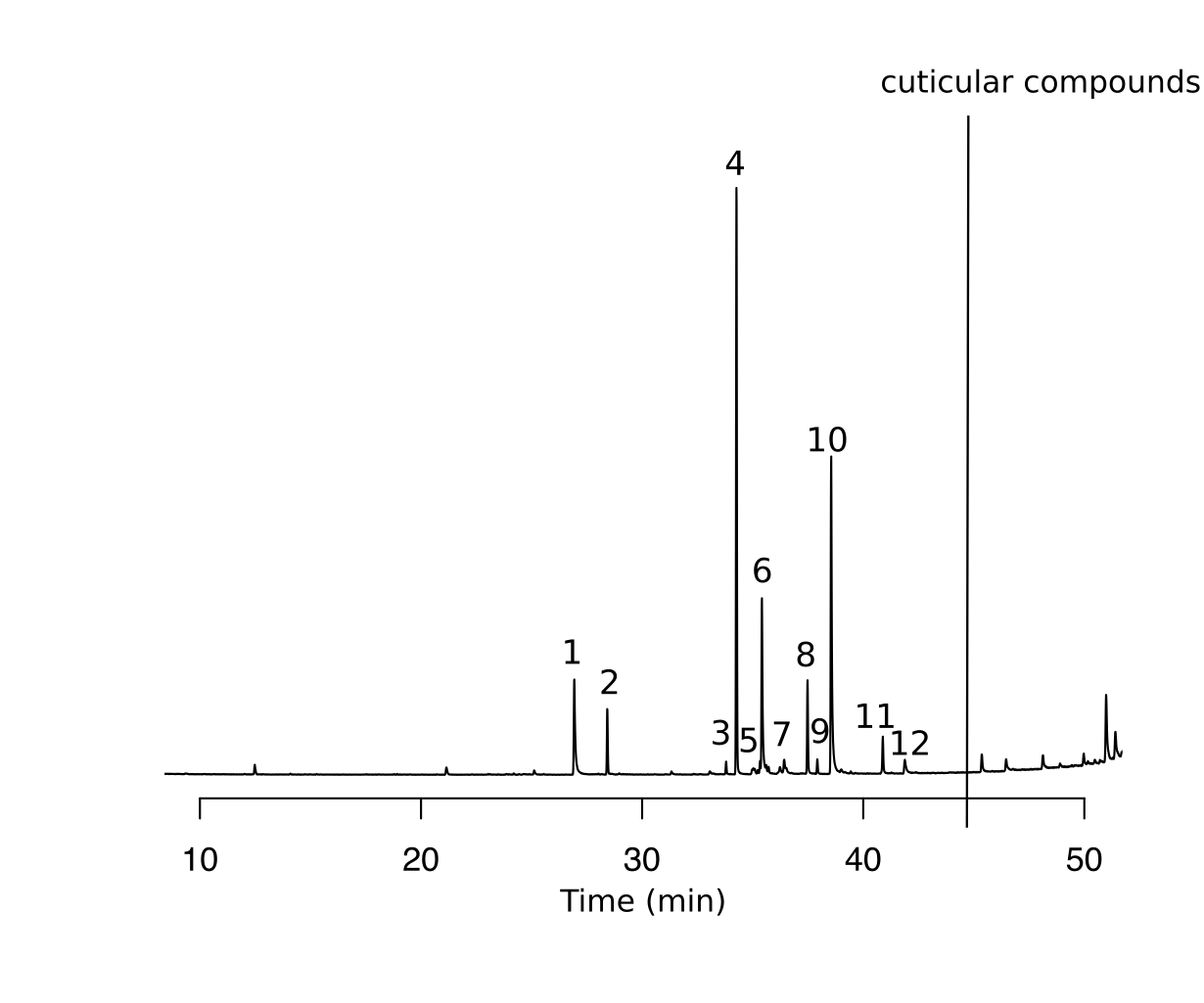


**Figure S1. Total ion chromatogram of extract from androconial region of *H. melpomene*. 1, Syringaldehyde; 2, internal standard; 3, (*Z*)-9-Octadecenal; 4, Octadecanal; 5, Methyloctadecanals; 6, 1-Octadecanol; 7, Methyloctadecan-1-ol; 8, (*Z*)-11-Icosenal; 9, Icosanal; 10, (*Z*)-11-Icosenol; 11, (*Z*)-13-Docosenal; 12, (*Z*)-13-Docosen-1-ol. All peaks later than 45 minutes are cuticular compounds. Abundances are scaled to the highest peak.**


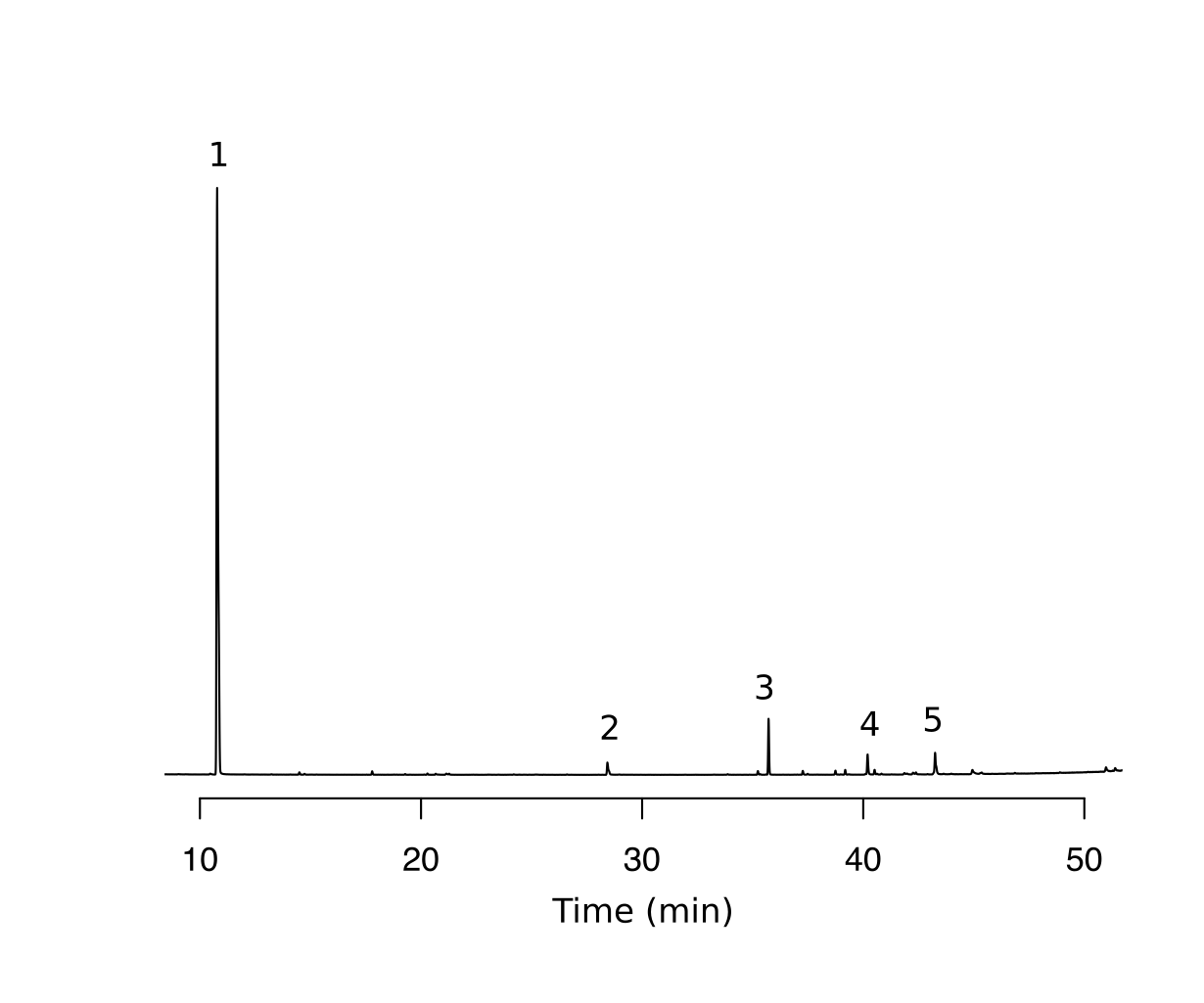


**Figure S2. Total ion chromatogram of extract from genital region of *H. melpomene*. 1, (*E*)-β-Ocimene; 2, internal standard; 3, Henicosane; 4, (*Z*)-3-Hexenyl hexadecanoate; 5, Hexyl octadecenoate & (*Z*)-3-Hexenyl octadecenoate. Abundances are scaled to the highest peak.**

**Table S1: Androconial compounds identified in *H. melpomene rosina* males reared on *P. platyloba* (19 individuals), *P. menispermifolia* (11 individuals) or *P. vitifolia* (12 individuals), with a mean amount greater than 1 ng in at least one of the treatments. The gas chromatographic retention index (RI) is reported for each compound. Mean amounts (ng) ± standard deviation, as well as false detection rate corrected p-values. Compounds in bold are predicted to be plant-derived.**

| **Chemical** | **RI** | ***P. platyloba*** | ***P. menispermifolia*** | ***P. vitifolia*** |  |
| --- | --- | --- | --- | --- | --- |
| *o*-Guaiacol | 1090 | 1.50±1.73 | 1.52±1.41 | 1.43±1.28 | NS |
| Nonanal | 1105 | 25.31±34.96 | 11.64±8.88 | 10.10±3.91 | NS |
| Benzoic acid | 1166 | 3.61±7.53 | 0.07±0.16 | 1.15±1.97 | NS |
| Naphthalene | 1181 | 1.22±1.13 | 2.17±1.05 | 1.06±0.65 | NS |
| Methyl salicylate | 1189 | 0.59±1.97 | 0.98±3.13 | 2.32±1.95 | 0.026 |
| Decanal | 1198 | 1.80±5.65 | 0.86±1.69 | 0.10±0.34 | NS |
| Unknown compound | 1353 | 0±0 | 0.40±1.31 | 3.30±3.85 | 0.006 |
| Dihydroactinidiolide | 1532 | 1.41±1.65 | 0.52±0.72 | 0.48±0.63 | NS |
| **Syringaldehyde** | 1662 | 641.31±407.13 | 406.92±228.15 | 442.11±229.66 | NS |
| **3,5-Dimethoxy-4-hydroxybenzyl alcohol** | 1707 | 3.67±6.85 | 1.34±4.45 | 1.72±2.24 | NS |
| Unknown compound | 1709 | 0.25±0.68 | 1.16±1.48 | 0.16±0.35 | NS |
| **1-(3,5-Dimethoxy-4-hydroxybenzyl)ethanone** | 1735 | 0.99±1.58 | 0.67±0.76 | 0.01±0.04 | 0.022 |
| Unknown hydrocarbon | 1753 | 1.53±2.08 | 3.46±2.87 | 1.01±1.72 | NS |
| Ethyl benzoate | 1762 | 2.02±1.99 | 1.37±1.14 | 1.52±1.03 | NS |
| Benzyl benzoate | 1766 | 0.25±0.57 | 0.13±0.44 | 1.27±1.40 | 0.022 |
| **1-(4-Hydroxy-3,5-dimethoxyphenyl)-2-propen-1-one** | 1807 | 1.48±1.93 | 1.15±1.32 | 0±0 | 0.016 |
| Methyl 1*H*-indol-3-acetate | 1822 | 3.37±3.20 | 3.28±2.79 | 2.10±2.63 | NS |
| Methyl 1*H*-indol-3-carboxylate | 1853 | 2.57±2.37 | 1.67±2.54 | 0.98±1.14 | NS |
| Benzyl salicylate | 1870 | 1.44±2.57 | 0.57±0.97 | 1.99±3.67 | NS |
| **Syringaldehyde derivative** | 1891 | 0±0 | 3.05±2.74 | 0±0 | 0.003 |
| Nonadecane | 1898 | 1.47±2.69 | 2.48±4.31 | 0.56±1.04 | NS |
| Unknown compound | 1914 | 1.27±1.80 | 1.33±1.11 | 0.78±0.86 | NS |
| Unknown hydrocarbon | 1962 | 1.32±1.14 | 1.80±1.41 | 0.28±0.97 | 0.022 |
| (*Z*)-9-Octadecenal | 1996 | 11.68±14.04 | 6.34±4.97 | 7.53±9.54 | NS |
| Octadecanal | 2021 | 739.57±422.66 | 617.88±536.70 | 482.05±390.61 | NS |
| **Ethyl 4-hydroxy-3,5- dimethoxybenzoate** | 2057 | 32.61±55.79 | 11.60±13.95 | 0.14±0.37 | 0.011 |
| Methyloctadecanal | 2065 | 8.11±5.99 | 5.69±4.96 | 2.56±3.81 | NS |
| Henicosadiene | 2065 | 1.36±4.28 | 0.22±0.74 | 2.97±3.91 | 0.030 |
| Methyloctadecanal | 2072 | 5.35±3.49 | 3.65±2.23 | 2.12±1.90 | NS |
| Methyloctadecanal | 2077 | 30.77±15.99 | 15.37±8.54 | 10.94±13.15 | 0.011 |
| Octadecan-1-ol | 2082 | 231.72±153.72 | 660.33±861.35 | 224.31±240.45 | NS |
| (Z)-16-Methyl-9-octadecenol | 2092 | 4.97±4.17 | 5.67±4.76 | 2.80±3.43 | NS |
| Henicosane | 2099 | 11.29±15.86 | 15.19±21.48 | 9.49±8.28 | NS |
| Unknown alkene or alcohol | 2127 | 16.38±7.55 | 16.65±13.98 | 10.54±6.22 | NS |
| Unknown compound | 2132 | 36.70±19.06 | 45.70±35.74 | 24.82±14.49 | NS |
| Methyloctadecan-1-ol | 2138 | 9.30±6.07 | 6.09±8.66 | 6.25±5.97 | NS |
| (*Z*)-11-Icosenal | 2198 | 168.51±128.84 | 62.40±46.48 | 70.95±57.40 | NS |
| Icosanal | 2224 | 23.21±11.46 | 16.08±14.41 | 8.41±7.59 | 0.009 |
| (*Z*)-11-Icosenol | 2261 | 619.09±311.94 | 514.62±436.15 | 465.59±229.14 | NS |
| Tricosene | 2281 | 3.98±3.12 | 8.03±8.92 | 2.73±2.85 | NS |
| Tricosane | 2297 | 1.19±2.43 | 1.16±1.30 | 0.69±1.02 | NS |
| Fatty acid amide | 2325 | 0.27±1.20 | 0±0 | 1.09±1.66 | 0.015 |
| Unknown amide | 2374 | 2.17±6.01 | 0±0 | 2.04±4.82 | NS |
| Unknown compound | 2392 | 12.30±18.70 | 5.30±10.87 | 1.23±2.86 | NS |
| (*Z*)-13-Docosenal | 2403 | 66.30±54.95 | 32.82±40.35 | 32.03±25.53 | NS |
| (*Z*)-13-Docosen-1-ol | 2464 | 31.80±22.13 | 53.03±93.01 | 35.28±30.98 | NS |
| Pentacosane | 2500 | 3.43±4.69 | 1.86±1.84 | 2.42±1.77 | NS |

**Table S2: Genital compounds identified in *H. melpomene rosina* males reared on *P. platyloba* (17 individuals), *P. menispermifolia* (13 individuals) or *P. vitifolia* (13 individuals), with a mean amount greater than 1 ng in at least one of the treatments. The gas chromatographic retention index (RI) is reported for each compound. Mean amounts (ng) ± standard deviation, as well as false detection rate corrected p-values. Compounds in bold are predicted to be plant-derived.**

| **Chemical** | | **RI** | ***P. platyloba*** | ***P. menispermifolia*** | ***P. vitifolia*** |  |
| --- | --- | --- | --- | --- | --- | --- |
| β-Myrcene | 990 | | 11.94±13.62 | 15.16±6.25 | 19.34±31.48 | NS |
| (*Z*)-β-Ocimene | 1030 | | 2822.73±11451.08 | 70.18±27.71 | 246.54±635.68 | NS |
| (*E*)-β-Ocimene | 1054 | | 34789.16±22151.25 | 35742.69±9716.58 | 39065.11±30676.99 | NS |
| *o*-Guaiacol | 1090 | | 0.33±0.67 | 0.34±0.75 | 1.08±1.81 | NS |
| Alloocimene | 1129 | | 8.64±11.64 | 14.34±14.11 | 26.66±30.00 | NS |
| 2-sec-Butyl-3-methoxypyrazine | 1173 | | 41.38±21.95 | 51.89±27.36 | 46.52±27.05 | NS |
| 2-Methoxy-3-isobutylpyrazine | 1181 | | 15.45±9.53 | 19.99±12.12 | 17.55±8.85 | NS |
| Methyl salicylate | 1189 | | 0.26±1.05 | 0.63±2.14 | 1.15±2.22 | NS |
| Dihydroedulan II | 1290 | | 35.91±29.64 | 14.78±11.83 | 23.38±25.46 | NS |
| **7-β-(*H*)-Silphiperfol-5-ene** | 1345 | | 9.75±12.23 | 0±0 | 0±0 | <0.001 |
| **8-Hydroxylinalool** | 1349 | | 0±0 | 0±0 | 4.07±7.10 | NS |
| **Unknown sesquiterpene** | 1378 | | 2.87±4.20 | 0±0 | 0±0 | 0.001 |
| **Unknown sesquiterpene** | 1384 | | 20.90±21.99 | 0±0 | 0±0 | <0.001 |
| Unknown compound | 1396 | | 39.76±27.38 | 26.83±30.96 | 8.23±6.87 | 0.004 |
| **β-Caryophyllene** | 1417 | | 18.97±22.09 | 0±0 | 0±0 | <0.001 |
| 11-Dodecanolide | 1465 | | 2.86±5.50 | 4.34±7.34 | 3.84±9.72 | NS |
| 14-Tetradecanolide | 1733 | | 9.64±19.18 | 0±0 | 0±0 | NS |
| Ethyl benzoate | 1762 | | 2.18±2.14 | 1.56±2.63 | 1.50±3.99 | NS |
| Hexadecenolide | 1850 | | 0.27±1.13 | 0±0 | 1.36±3.37 | NS |
| 15-Hexadecanolide | 1855 | | 0.18±0.54 | 2.87±5.59 | 0.82±2.09 | NS |
| Hexadecenolide | 1865 | | 4.29±13.80 | 1.28±4.61 | 1.53±5.52 | NS |
| (*Z*)-9-Octadecenal | 1996 | | 0±0 | 0±0 | 5.35±1.13 | NS |
| Icosane | 1998 | | 10.14±12.67 | 12.07±6.51 | 10.97±11.86 | NS |
| Octadecadienolide | 2033 | | 0.36±1.01 | 2.37±5.95 | 4.27±12.07 | NS |
| (Z)-9-Octadecen-11-olide | 2038 | | 5.88±19.37 | 2.40±8.66 | 1.20±4.33 | NS |
| (Z)-9- Octadecen -13-olide | 2044 | | 5.17±19.30 | 2.24±5.50 | 4.73±17.06 | NS |
| Octadecanoic acid ester | 2047 | | 1.67±3.97 | 6.21±11.12 | 2.10±5.94 | NS |
| Unknown macrolide | 2048 | | 1.16±2.28 | 4.68±6.25 | 0.53±1.30 | NS |
| Henicosene | 2068 | | 53.38±54.05 | 51.03±29.18 | 74.85±122.55 | NS |
| Henicosene | 2072 | | 11.49±16.42 | 10.88±8.71 | 17.30±21.31 | NS |
| Henicosene | 2086 | | 5.98±8.64 | 5.89±5.16 | 5.41±11.25 | NS |
| Henicosane | 2100 | | 1614.47±1165.36 | 1569.21±536.05 | 1502.11±1289.54 | NS |
| 9-Octadecen-18-olide | 2123 | | 0.69±2.08 | 16.44±29.73 | 5.79±14.62 | NS |
| 18-Octadecanolide | 2135 | | 2.62±4.86 | 4.98±7.66 | 5.47±13.83 | NS |
| Butyl hexadecenoate | 2153 | | 5.63±14.40 | 1.76±4.62 | 3.28±8.21 | NS |
| Ethyl oleate | 2160 | | 2.22±6.34 | 5.64±14.76 | 83.31±245.15 | NS |
| Butyl hexadecanoate | 2186 | | 146.79±189.69 | 73.69±61.43 | 152.44±235.08 | NS |
| Isopropyl oleate | 2188 | | 8.21±26.61 | 89.25±129.52 | 82.97±156.19 | NS |
| Docosane | 2198 | | 21.18±20.35 | 26.90±22.85 | 15.24±16.54 | NS |
| Unknown compound | 2250 | | 0±0 | 1.48±5.32 | 1.33±2.09 | 0.039 |
| Tricosene | 2270 | | 84.15±83.76 | 102.54±78.73 | 180.70±300.84 | NS |
| Tricosene | 2275 | | 30.83±54.18 | 21.16±24.96 | 16.24±14.90 | NS |
| Isopentyl ester | 2280 | | 0.16±0.64 | 5.89±18.52 | 6.00±17.52 | NS |
| Isobutyl oleate | 2287 | | 19.14±50.51 | 264.92±578.20 | 342.60±833.26 | NS |
| Tricosane | 2298 | | 140.17±936.38 | 126.64±107.42 | 129.47±123.82 | NS |
| Unknown compound | 2305 | | 6.85±11.73 | 2.72±5.56 | 4.78±12.70 | NS |
| Hexyl hexadecenoate | 2353 | | 4.74±17.52 | 9.25±33.36 | 2.33±8.39 | NS |
| Butyl oleate | 2355 | | 845.44±1032.66 | 1028.79±939.16 | 1213.21±1386.81 | NS |
| Butyl octadecadienoate | 2355 | | 28.30±65.79 | 22.08±39.32 | 28.95±44.74 | NS |
| (*Z*)-3-Hexenyl hexadecanoate | 2379 | | 288.69±344.78 | 297.39±297.88 | 217.63±310.39 | NS |
| Butyl octadecanoate | 2386 | | 58.67±119.39 | 26.76±30.34 | 55.17±92.62 | NS |
| Isoprenyl octadec-11-enoate | 2436 | | 5.15±18.52 | 3.17±6.66 | 12.65±32.87 | NS |
| Unknown compound | 2460 | | 2.25±9.26 | 2.78±10.03 | 19.24±52.08 | NS |
| (*Z*)-13-Docosen-1-ol | 2464 | | 69.50±115.09 | 55.06±32.28 | 70.20±114.92 | NS |
| Unknown compound | 2466 | | 4.15±17.10 | 7.10±13.76 | 0±0 | NS |
| 1-Docosanol | 2489 | | 408.14±706.49 | 399.90±295.30 | 486.75±712.49 | NS |
| Pentacosane | 2500 | | 60.51±67.58 | 53.40±35.11 | 57.50±55.56 | NS |
| 11-Methylpentacosane | 2532 | | 20.72±28.09 | 13.23±15.59 | 18.77±29.49 | NS |
| Unknown compound | 2550 | | 61.86±157.89 | 42.53±66.66 | 51.60±104.67 | NS |
| Hexyl octadecenoate and (Z)-3-hexenyl octadecenoate | 2557 | | 1398.41±1699.54 | 1724.97±1123.13 | 1689.16±2098.32 | NS |
| Hexenyl octadecatrienoate and (Z)-3-Hexenyl octadecatrienoate | 2561 | | 216.83±278.88 | 271.38±240.37 | 323.64±504.26 | NS |
| (Z)-3-Hexenyl octadecanoate | 2581 | | 94.71±171.44 | 88.06±108.84 | 84.38±148.61 | NS |
| Hexacosane | 2600 | | 2.51±6.33 | 0±0 | 1.08±3.88 | NS |
| 1,3-Docosanediol cyclic dimethylsilyl- derivative | 2604 | | 128.18±210.59 | 208.91±125.60 | 164.30±355.66 | NS |
| Tetracosenol | 2670 | | 601.62±936.38 | 762.63±414.84 | 1038.29±1582.15 | NS |
| 1-Tetracosanol | 2694 | | 226.72±435.64 | 335.14±296.89 | 366.72±439.37 | NS |
| Unknown compound | 2694 | | 0±0 | 6.35±22.89 | 445.39±1236.54 | NS |
| Heptacosane | 2700 | | 131.7±210.29 | 61.09±47.31 | 55.22±82.73 | NS |
| 11-Methylheptacosane | 2725 | | 12.99±25.46 | 6.91±8.16 | 4.73±9.83 | NS |
| Cholestadiene | 2744 | | 0±0 | 9.56±34.48 | 18.18±24.10 | <0.001 |
| Unknown compound | 2746 | | 1.90±7.30 | 0.12±0.44 | 1.84±5.05 | NS |
| Tetracosenolide | 2749 | | 0±0 | 13.03±22.58 | 4.05±14.51 | NS |
| Unknown compound | 2750 | | 0±0 | 5.09±11.07 | 0±0 | NS |
| Unknown compound | 2752 | | 5.41±15.64 | 0±0 | 3.51±12.66 | NS |
| Unknown compound | 2753 | | 1.92±7.62 | 1.82±6.56 | 0±0 | NS |
| Unknown compound | 2770 | | 63.97±102.60 | 332.49±523.57 | 94.07±153.78 | NS |
| Unknown compound | 2783 | | 7.03±20.83 | 0±0 | 8.64±31.13 | NS |
| 1,3-Tetracosanediol cyclic dimethylsilyl- derivative | 2799 | | 225.11±454.13 | 363.47±270.74 | 363.61±816.74 | NS |
| Unknown compound | 2822 | | 4.08±14.37 | 0.62±2.22 | 0±0 | NS |
| Hexacosanal | 2829 | | 62.94±125.32 | 64.13±51.46 | 158.10±331.50 | NS |

**Table S3: Pairwise comparisons of dispersion of *H. melpomene* genital compounds when reared on different plants. (Observed p-value below diagonal, permuted p-value above diagonal).**

|  | *P. menispermifolia* | *P. platyloba* | *P. vitifolia* |
| --- | --- | --- | --- |
| *P. menispermifolia* |  | 0.002 | 0.021 |
| *P. platyloba* | 0.007 |  | 0.964 |
| *P. vitifolia* | 0.024 | 0.962 |  |

**Table S4: Androconial compounds identified in *H. melpomene rosina* males fed as adults with (20 individuals) or without pollen (33 individuals) with a mean amount greater than 1 ng in at least one of the treatments. The gas chromatographic retention index (RI) is reported for each compound. Mean amounts (ng) ± standard deviation. No differences were found to be statistically significant.**

| **Chemical** | **RI** | **With pollen** | **Without pollen** |
| --- | --- | --- | --- |
| Nonanal | 1105 | 20.02±6.30 | 28.38±11.46 |
| Benzoic acid | 1166 | 8.82±8.46 | 7.18±6.99 |
| Naphthalene | 1181 | 2.26±1.36 | 1.94±2.50 |
| Homovanillyalcohol | 1523 | 1.26±1.71 | 0.98±2.08 |
| Syringaldehyde | 1662 | 592.20±296.89 | 501.25±230.87 |
| 3,5-Dimethoxy-4-hydroxybenzyl alcohol | 1707 | 3.90±4.40 | 2.71±3.68 |
| 1-(3,5-Dimethoxy-4-hydroxybenzyl)ethanone | 1735 | 4.17±3.54 | 5.83±5.09 |
| Unknown hydrocarbon | 1750 | 2.32±2.72 | 2.42±3.42 |
| Ethyl benzoate | 1762 | 1.69±1.19 | 2.60±2.64 |
| Benzyl benzoate | 1766 | 2.28±2.99 | 0.67±1.29 |
| 1-(4-Hydroxy-3,5-dimethoxyphenyl)-2-propen-1-one | 1807 | 5.23±5.02 | 6.76±8.41 |
| Methyl 1*H*-indol-3-acetate | 1822 | 4.25±2.99 | 3.26±3.09 |
| Methyl 1*H*-indol-3-carboxylate | 1853 | 1.47±2.22 | 1.42±1.79 |
| Unknown compound | 1914 | 1.37±1.61 | 0.69±0.95 |
| Unknown compound | 1930 | 1.75±2.14 | 0.79±1.51 |
| Hexadecanoic acid | 1960 | 5.61±5.01 | 5.44±5.27 |
| (*Z*)-9-Octadecenal | 1996 | 10.69±7.61 | 9.24±8.44 |
| Octadecanal | 2021 | 688.33±388.77 | 626.43±327.87 |
| 9-Octadecen-1-ol | 2040 | 180.69±7.61 | 9.24±8.44 |
| Nonadecanal, methyl, branched | 2054 | 5.61±5.01 | 5.44±5.27 |
| Ethyl 4-hydroxy-3,5- dimethoxybenzoate | 2057 | 16.52±16.30 | 18.36±19.15 |
| Unknown compound | 2057 | 7.94±4.78 | 6.90±5.21 |
| Methyloctadecanal | 2065 | 8.13±6.03 | 7.32±7.39 |
| Henicosadiene | 2065 | 3.31±4.30 | 2.47±3.94 |
| Methyloctadecanal | 2072 | 6.56±5.21 | 5.56±5.27 |
| Methyloctadecanal | 2077 | 21.03±14.15 | 25.57±15.99 |
| Octadecan-1-ol | 2082 | 384.90±286.32 | 264.82±225.41 |
| (Z)-16-Methyl-9-octadecenol | 2092 | 4.60±6.29 | 5.91±5.08 |
| Henicosane | 2099 | 10.12±5.72 | 15.23±7.61 |
| Unknown compound | 2103 | 3.00±7.81 | 1.95±6.44 |
| Unknown compound | 2112 | 1.62±2.99 | 0.12±0.71 |
| Unknown alkene or alcohol | 2127 | 27.64±17.93 | 22.76±14.84 |
| Unknown compound | 2132 | 35.81±36.17 | 17.46±26.56 |
| Methyloctadecan-1-ol | 2138 | 17.35±11.25 | 17.10±10.78 |
| Unknown hydrocarbon | 2143 | 1.38±4.23 | 0.82±3.40 |
| Octadecanoic acid | 2160 | 1.79±3.09 | 0.54±1.79 |
| Unknown alcohol | 2166 | 1.08±2.24 | 1.21±2.44 |
| (*Z*)-11-Icosenal | 2198 | 103.12±70.73 | 105.61±77.15 |
| Icosanal | 2224 | 16.58±9.85 | 15.82±9.47 |
| (*Z*)-11-Icosenol | 2261 | 768.01±361.82 | 523.86±343.46 |
| Tricosene | 2281 | 7.76±6.12 | 6.19±5.45 |
| Fatty acid amide | 2325 | 2.29±30.1 | 2.80±3.93 |
| Unknown compound | 2352 | 6.03±12.03 | 3.72±6.66 |
| Unknown amide | 2374 | 2.34±8.40 | 3.96±19.32 |
| Unknown compound | 2392 | 4.41±6.26 | 7.88±34.56 |
| (*Z*)-13-Docosenal | 2403 | 44.77±34.60 | 35.19±30.69 |
| (*Z*)-13-Docosen-1-ol | 2464 | 60.82±52.24 | 33.23±41.49 |
| Unknown compound | 2465 | 1.78±4.71 | 4.09±6.46 |
| Pentacosane | 2500 | 10.57±9.64 | 7.70±6.69 |

**Table S5: Genital compounds identified in *H. melpomene rosina* males fed as adults with (20 individuals) or without pollen (27 individuals) with a mean amount greater than 1 ng in at least one of the treatments. The gas chromatographic retention index (RI) is reported for each compound. Mean amounts (ng) ± standard deviation. No differences were found to be statistically significant.**

| **Chemical** | **RI** | **With pollen** | **Without pollen** |
| --- | --- | --- | --- |
| β-Myrcene | 990 | 9.39±7.27 | 14.87±11.98 |
| (*Z*)-β-Ocimene | 1030 | 881.98±3725.80 | 971.89±4438.55 |
| (*E*)-β-Ocimene | 1054 | 21179.02±10155.09 | 30487.62±18517.35 |
| Nonanal | 1105 | 3.61±2.43 | 4.34±5.42 |
| Alloocimene | 1129 | 21.45±15.80 | 39.62±39.16 |
| (4*E*,6*Z*)-2,6-Dimethyl-2,4,6-octatriene | 1140 | 10.10±11.40 | 13.04±18.50 |
| 2-sec-Butyl-3-methoxypyrazine | 1173 | 25.95±20.86 | 25.81±20.67 |
| 2-Methoxy-3-isobutylpyrazine | 1181 | 10.61±8.27 | 9.59±5.70 |
| Napthalene | 1181 | 1.32±1.56 | 4.34±7.71 |
| Methyl salicylate | 1189 | 4.51±9.46 | 5.35±10.85 |
| Dihydroedulan II | 1290 | 8.78±11.13 | 15.20±14.38 |
| Unknown sesquiterpene | 1384 | 6.30±11.42 | 12.43±23.95 |
| Unknown compound | 1396 | 16.07±13.93 | 26.85±20.84 |
| β-Caryophyllene | 1417 | 3.88±10.48 | 8.91±29.30 |
| Unknown compound | 1433 | 0.84±2.05 | 2.36±4.05 |
| 11-Dodecanolide | 1465 | 6.73±9.05 | 8.40±9.69 |
| 14-Tetradecanolide | 1733 | 11.74±24.63 | 4.47±11.99 |
| Ethyl benzoate | 1762 | 1.44±1.62 | 1.57±2.09 |
| Hexadecenolide | 1845 | 5.53±17.19 | 9.55±24.97 |
| Unknown compound | 1859 | 0.79±3.19 | 1.21±5.21 |
| Nonadecane | 1899 | 1.61±3.35 | 1.42±3.36 |
| Hexadecanoic acid | 1960 | 15.85±19.88 | 17.87±29.71 |
| (*Z*)-9-Octadecenal | 1996 | 7.50±10.06 | 6.10±12.93 |
| Icosane | 1998 | 18.11±19.35 | 19.47±19.80 |
| Octadecadienolide | 2035 | 3.31±4.86 | 1.31±2.96 |
| (Z)-9- Octadecen -13-olide | 2044 | 7.16±17.20 | 29.61±71.06 |
| Octadecanoic acid ester | 2047 | 8.75±17.77 | 14.61±35.16 |
| Unknown macrolide | 2048 | 3.77±8.21 | 4.27±11.31 |
| Henicosene | 2068 | 84.17±89.07 | 82.57±111.40 |
| Henicosene | 2072 | 40.57±27.09 | 47.44±29.12 |
| Henicosene | 2086 | 14.82±16.54 | 12.33±14.01 |
| Henicosane | 2100 | 1673.83±1185.53 | 2040.62±1566.25 |
| 9-Octadecen-18-olide | 2123 | 11.57±23.92 | 17.70±42.81 |
| 18-Octadecanolide | 2135 | 13.74±18.73 | 25.94±35.28 |
| Butyl hexadecenoate | 2153 | 25.92±57.62 | 15.67±49.62 |
| Ethyl oleate | 2160 | 147.50±457.41 | 10.19±35.77 |
| Butyl hexadecanoate | 2186 | 290.97±395.89 | 263.52±358.96 |
| Isopropyl oleate | 2188 | 66.81±130.57 | 202.02±428.01 |
| Docosane | 2198 | 26.73±24.06 | 26.97±27.21 |
| Icosanal | 2228 | 4.45±4.07 | 10.26±12.09 |
| Isoprenyl palmitate | 2254 | 3.10±6.48 | 4.32±17.03 |
| Fatty acid amide | 2325 | 1.07±2.70 | 3.92±9.83 |
| Unknown compound | 2250 | 1.97±2.93 | 2.73±4.94 |
| Tricosene | 2270 | 127.52±118.14 | 174.15±312.73 |
| Tricosene | 2275 | 53.20±33.49 | 239.55±784.00 |
| Isopentyl ester | 2280 | 2.93±6.25 | 34.07±115.31 |
| Isobutyl oleate | 2287 | 272.35±494.86 | 456.07±1058.14 |
| Tricosane | 2298 | 151.85±140.12 | 145.52±150.42 |
| Unknown compound | 2305 | 25.00±50.08 | 17.72±30.82 |
| Butyl oleate | 2355 | 2214.19±2368.51 | 2678.85±2991.26 |
| Butyl octadecadienoate | 2355 | 63.55±84.53 | 117.52±197.94 |
| (*Z*)-3-Hexenyl hexadecanoate | 2379 | 346.46±490.37 | 280.53±356.56 |
| Butyl octadecanoate | 2386 | 94.73±130.80 | 103.26±153.70 |
| Tetracosane | 2400 | 3.31±4.86 | 1.31±2.96 |
| 19-Methyleicosyl acetate | 2422 | 5.31±11.84 | 6.75±14.63 |
| Unknown aldehyde | 2422 | 9.51±7.39 | 7.60±9.96 |
| Isoprenyl octadec-11-enoate | 2436 | 30.34±51.83 | 66.75±177.60 |
| (*Z*)-13-Docosen-1-ol | 2464 | 89.49±117.01 | 173.83±219.21 |
| 1-Docosanol | 2489 | 496.28±431.58 | 881.27±900.58 |
| Pentacosane | 2500 | 137.90±141.25 | 100.48±84.41 |
| 11-Methylpentacosane | 2532 | 66.03±83.60 | 32.51±38.54 |
| Hexyl octadecadienoate | 2544 | 33.98±130.16 | 8.12±40.30 |
| Hexyl octadecenoate and (*Z*)-3-hexenyl octadecenoate | 2557 | 2058.15±2294.58 | 3106.82±3582.23 |
| Hexenyl octadecatrienoate and (*Z*)-3-hexenyl octadecatrienoate | 2561 | 309.53±337.68 | 364.39±579.66 |
| (*Z*)-3-Hexenyl octadecanoate | 2581 | 140.28±188.85 | 161.72±240.14 |
| Hexyl octadecanoate | 2590 | 77.96±152.82 | 198.52±347.46 |
| Hexacosane | 2600 | 29.70±30.06 | 13.90±28.32 |
| 1,3-Docosanediol cyclic dimethylsilyl-derivative | 2604 | 119.97±115.65 | 327.36±499.90 |
| Tetracosenol | 2670 | 786.60±733.25 | 1626.51±1667.51 |
| 1-Tetracosanol | 2694 | 491.61±567.03 | 1108.74±1589.39 |
| Heptacosane | 2700 | 149.12±88.41 | 203.20±138.24 |
| 11-Methylheptacosane | 2725 | 29.63±30.91 | 51.28±39.67 |
| Unknown compound | 2746 | 7.77±16.27 | 0±0 |
| Unknown compound | 2770 | 201.71±348.54 | 174.97±163.95 |
| Unknown compound | 2783 | 12.57±22.04 | 40.47±57.57 |
| 1,3-Tetracosanediol cyclic dimethylsilyl-derivative | 2799 | 153.20±166.52 | 405.72±479.21 |
| Octacosane | 2800 | 11.58±21.23 | 7.52±15.09 |
| Hexacosanal | 2829 | 65.41±66.25 | 247.52±388.10 |
